# Supplementary figures and images for: Emission factors for Vietnamese beef cattle manure sun-drying and the effects of drying on manure microbial community
Source: PLoS One. 2022 Mar 16;17(3):e0264228. doi: 10.1371/journal.pone.0264228 (PMC8926181; doi:10.1371/journal.pone.0264228)

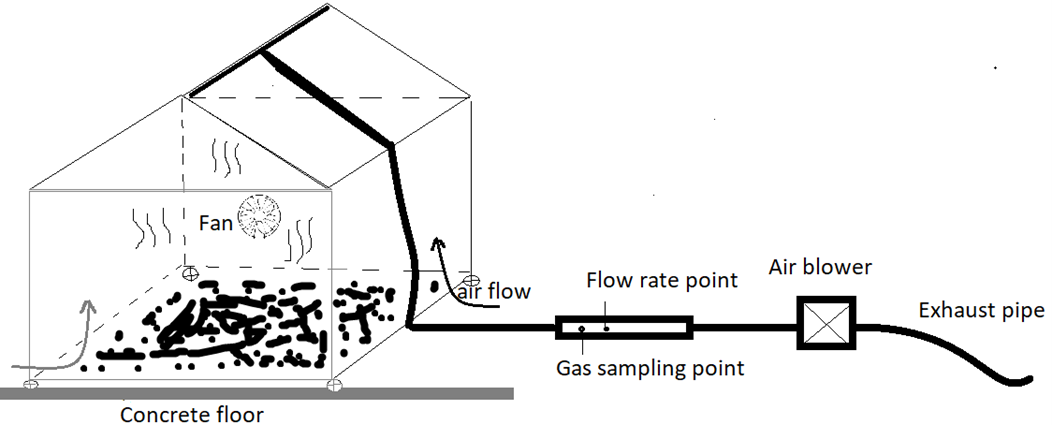

Supplement: S1 Fig — (TIF) [file pone.0264228.s001.tif]

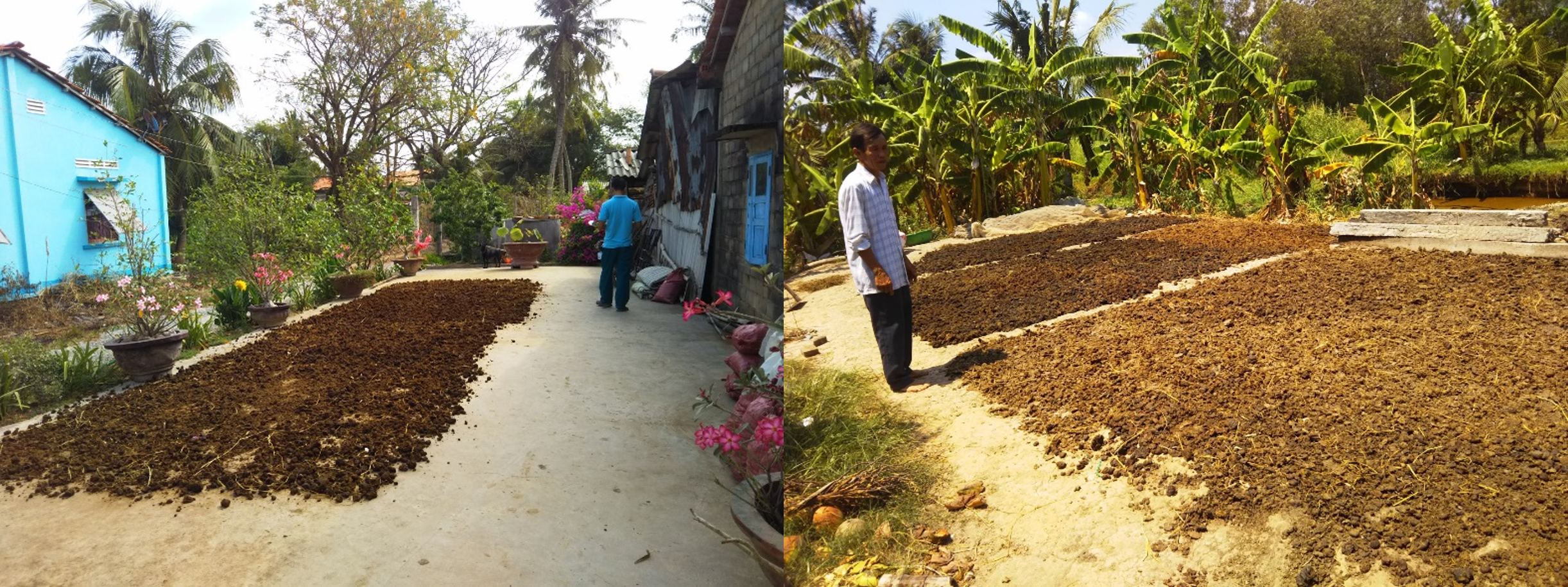

Supplement: S2 Fig — (TIF) [file pone.0264228.s002.tif]

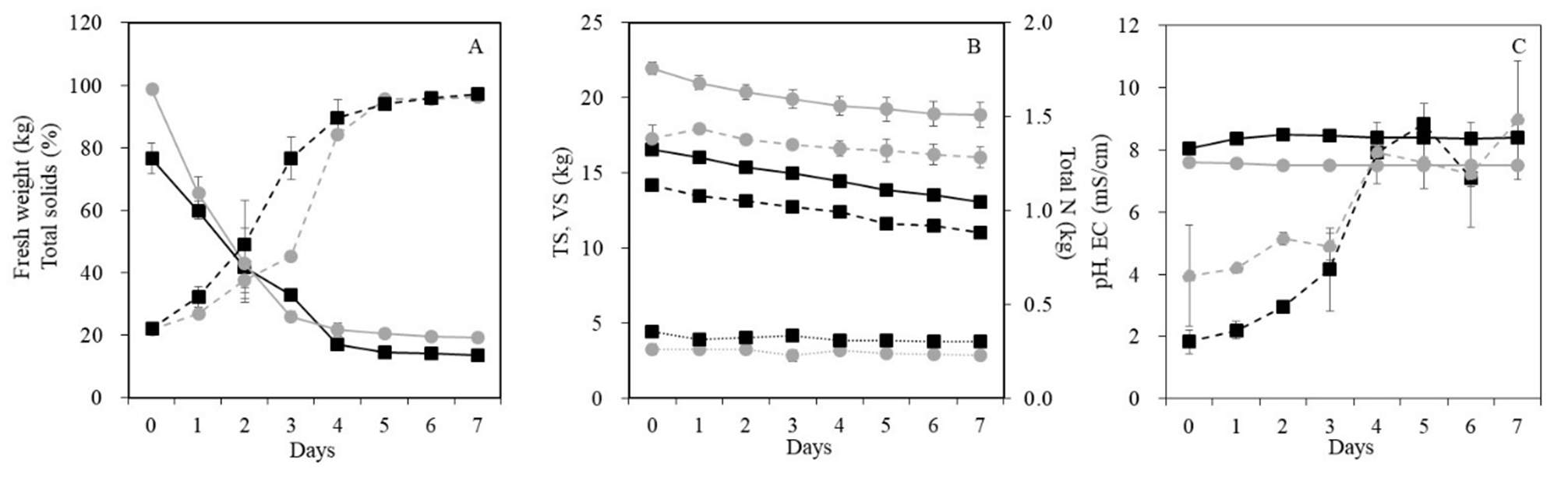

Supplement: S3 Fig — A: Weight, solid lines; total solids (TS), dashed lines. B: Total solids (TS), solid lines; volatile solids (VS), dashed lines; total nitrogen (N), dotted lines. C: pH, solid lines; electrical conductivity (EC), dashed lines. Black squares: Run 1. Gray circles: Run 2. Error bars: standard deviation (SD) (n = 2). (TIF) [file pone.0264228.s003.tif]

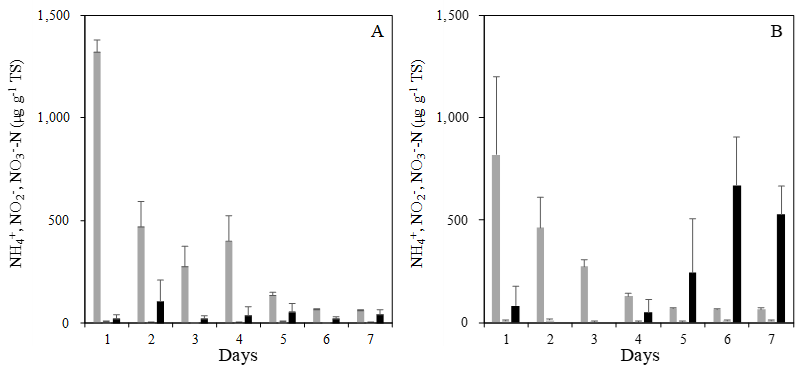

Supplement: S4 Fig — Error bars: SD (n = 2). TS: total solids. (TIF) [file pone.0264228.s004.tif]

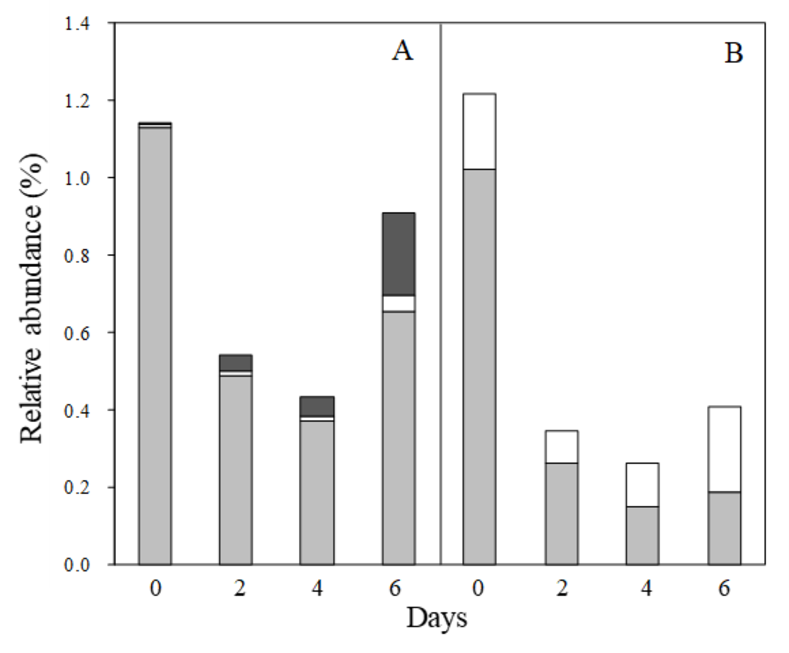

Supplement: S5 Fig — A and B indicate runs 1 and 2, respectively. Light gray bars: Methanobacteriales. Dark gray bars: Methanosarcinales. White bars: Methanomicrobiales. (TIF) [file pone.0264228.s005.tif]

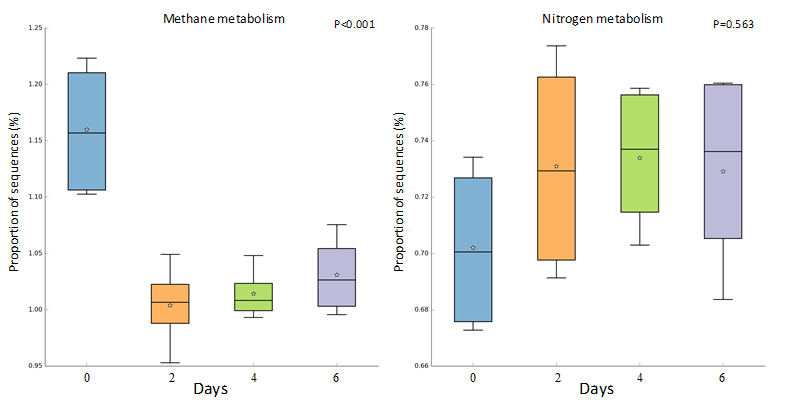

Supplement: S6 Fig — (TIF) [file pone.0264228.s006.tif]
