# Supplementary material for: Emission factors for Vietnamese beef cattle manure sun-drying and the effects of drying on manure microbial community
Source: PLoS One. 2022 Mar 16;17(3):e0264228. doi: 10.1371/journal.pone.0264228 (PMC8926181; doi:10.1371/journal.pone.0264228)
Supplement: S1 Table — (DOCX) [file pone.0264228.s007.docx]

| **S1 Table.** Primers sequence and PCR conditions for 16S rRNA, AOB*-amoA*, AOA-*amoA*, *mcrA*, *nirK*, *nirS* and *nosZ* | | | | | |  |
| --- | --- | --- | --- | --- | --- | --- |
| **Primer name** | **Sequence** | **Denature** | **Annealing** | **Elongation** | **Cycle time** | **Reference** |
| 341F | CCT ACG GGA GGC AGC AG | 95°C 10 sec | − | 62°C 34 sec | 30 | Muyzer et al., 1993 |
| 517R | ATT ACC GCG GCT GCT GG |  |  |  |  |  |
| amoA-1F | GGG GTT TCT ACT GGT GGT | 95°C 10 sec | 55°C 10 sec | 72°C 1 min | 40 | Rotthauwe et al., 1997 |
| amoA-2R | CCC CTC KGS AAA GCC TTC TTC |  |  |  |  |  |
| GenAOA-f | ATA GAG CCT CAA GTA GGA AAG TTC TA | 95°C 10 sec | 55°C 10 sec | 72°C 1 min | 40 | Meinhardt et al., 2015 |
| GenAOA-r | CCA AGC GGC CAT CCA GCT GTA TGT CC |  |  |  |  |  |
| mcrA-f | GGY GGT GTM GGD TTC ACM CAR TA | 95°C 10 sec | 62°C 10 sec | 72°C 30 sec | 40 | Angel et al., 2011 |
| mcrA-r | CGT TCA TBG CGT AGT TVG GRT AGT |  |  |  |  |  |
| F1aCu | ATC ATG GTS CTG CCG CG | 95°C 10 sec | 62°C 10 sec | 72°C 1 min | 40 | Throbäck et al., 2004 |
| R3Cu | GCC TCG ATC AGR TTG TGG TT |  |  |  |  |  |
| cd3aF | GTS AAC GTS AAG GAR ACS GG | 95°C 10 sec | 50°C 10 sec | 72°C 1 min | 40 | Throbäck et al., 2004 |
| R3cd | GAS TTC GGR TGS GTC TTG A |  |  |  |  |  |
| nosZ-F | CGY TGT TCM TCG ACA GCC AG | 95°C 10 sec | 62°C 10 sec | 72°C 1 min | 40 | Throbäck et al., 2004 |
| nosZ-R | CGS ACC TTS TTG CCS TYG CG |  |  |  |  |  |
