# Supplementary material for: Emission factors for Vietnamese beef cattle manure sun-drying and the effects of drying on manure microbial community
Source: PLoS One. 2022 Mar 16;17(3):e0264228. doi: 10.1371/journal.pone.0264228 (PMC8926181; doi:10.1371/journal.pone.0264228)
Supplement: S2 Table — (DOCX) [file pone.0264228.s008.docx]

| **S2 Table.** Chemical composition of feeds of the beef cattle in Bentre province | | | | | |
| --- | --- | --- | --- | --- | --- |
|  | **DM** | **OM** | **CP** | **EE** | **NDF** |
|  | **%** | **%DM** | **%DM** | **%DM** | **%DM** |
| Elephant grass | 15.7 | 85.8 | 10.9 | 4.0 | 66.9 |
| Para grass | 17.3 | 87.1 | 11.5 | 4.8 | 63.3 |
| Natural grass | 17.8 | 90.0 | 9.6 | 4.3 | 68.6 |
| Rice straw | 92.7 | 89.3 | 5.4 | 2.5 | 68.7 |
| Broken rice | 86.4 | 98.8 | 8.0 | 2.0 | 6.3 |
| Maize stover | 22.9 | 92.5 | 4.6 | 2.3 | 66.5 |
| Rice bran | 89.8 | 89.6 | 12.1 | 9.2 | 27.3 |
| White coconut meal | 89.3 | 95.6 | 20.0 | 10.4 | 52.7 |
| Brown coconut meal | 92.3 | 94.8 | 19.7 | 9.9 | 57.1 |
| Concentrate | 88.9 | 91.5 | 16.1 | 5.7 | 27.6 |
| CP: crude protein, DM: dry matter, EE: ethyl extract, NDF: neutral detergent fiber, OM: organic matter. | | | | | |
|  |  |  |  |  |  |
