# Supplementary material for: Emission factors for Vietnamese beef cattle manure sun-drying and the effects of drying on manure microbial community
Source: PLoS One. 2022 Mar 16;17(3):e0264228. doi: 10.1371/journal.pone.0264228 (PMC8926181; doi:10.1371/journal.pone.0264228)
Supplement: S3 Table — (DOCX) [file pone.0264228.s009.docx]

| **S3 Table.** Bacterial diversity during sun-drying of Vietnamese beef cattle manure | | | | | |
| --- | --- | --- | --- | --- | --- |
|  | **Day** | | | | **p-value** |
|  | **0** | **2** | **4** | **6** |  |
| Berger-Parker | 0.080^a^ | 0.050^b^ | 0.040^b^ | 0.049^b^ | 0.0063 |
| Brillouin | 5.399 | 5.237 | 5.379 | 5.355 | 0.5724 |
| Chao1 | 917.5 | 743.6 | 798.9 | 826.5 | 0.1229 |
| Evenness | 0.796 | 0.796 | 0.809 | 0.800 | 0.6789 |
| Faith's pd | 46.39 | 50.15 | 53.61 | 53.12 | 0.1716 |
| Simpson | 0.984 | 0.987 | 0.989 | 0.988 | 0.1891 |
| Shannon | 7.824 | 7.586 | 7.791 | 7.756 | 0.5671 |
