# Supplementary material for: Emission factors for Vietnamese beef cattle manure sun-drying and the effects of drying on manure microbial community
Source: PLoS One. 2022 Mar 16;17(3):e0264228. doi: 10.1371/journal.pone.0264228 (PMC8926181; doi:10.1371/journal.pone.0264228)
Supplement: S4 Table — (DOCX) [file pone.0264228.s010.docx]

| S4 Table Change of the manure chemical property during sun-drying experiment | | | | | | | | | | | | | | | | |  |  |  |
| --- | --- | --- | --- | --- | --- | --- | --- | --- | --- | --- | --- | --- | --- | --- | --- | --- | --- | --- | --- |
| Period | Run | FW (kg) | | TS (%) | | VS(%TS) | | TKN (%TS) | | pH | | EC (mS/cm) | | NH_4_^+^-N (µg/gTS) | | NO_2_^-^-N (µg/gTS) | | NO_3_^-^-N (µg/gTS) | |
|  |  | average | sd | average | sd | average | sd | average | sd | average | sd | average | sd | average | sd | average | sd | average | sd |
| Initial (day 0) | 1 | 76.5 | 5.0 | 21.7 | 1.3 | 85.7 | 0.2 | 1.56 | 0.04 | 8.1 | 0.1 | 1.8 | 0.4 | 1,321.4 | 57.0 | 5.2 | 4.0 | 20.9 | 19.0 |
| Final (day 7) | 1 | 13.6 | 0.3 | 96.2 | 0.5 | 84.5 | 0.5 | 1.74 | 0.04 | 8.4 | 0.0 | 7.1 | 0.3 | 61.9 | 2.8 | 3.3 | 0.9 | 42.9 | 22.7 |
| Initial (day 0) | 2 | 100.0 | 0.0 | 22.2 | 0.4 | 78.7 | 2.6 | 1.62 | 0.16 | 7.6 | 0.0 | 4.7 | 0.6 | 821.3 | 379.5 | 12.6 | 3.3 | 82.3 | 98.0 |
| Final (day 7) | 2 | 19.4 | 0.8 | 97.2 | 0.1 | 85.1 | 0.1 | 1.60 | 0.20 | 7.5 | 0.0 | 9.4 | 1.3 | 64.6 | 11.1 | 11.7 | 0.8 | 528.1 | 136.7 |
| FW, fresh weight; TS, total solids; VS, volatile solids; EC, electrical conductivity; TKN, total Kjeldahl nitrogen | | | | | | | | | | | | | | |  |  |  |  |  |
