# Supplementary material for: Emission factors for Vietnamese beef cattle manure sun-drying and the effects of drying on manure microbial community
Source: PLoS One. 2022 Mar 16;17(3):e0264228. doi: 10.1371/journal.pone.0264228 (PMC8926181; doi:10.1371/journal.pone.0264228)
Supplement: Attachment — Submitted filename: Emission factors for Vietnamese beef cattle manure sun-drying and the effects of dought stress on manure microbial community.docx [file pone.0264228.s011.docx]

Specific Suggestions:

Line 43: Are you saying Vietnam ranks third *in beef production* among the Association of South-East Asian Nations?

Line 93: Did you have 100% participation from those asked to complete the survey? How did you choose the 20 farms you selected? Do you feel this is representative of approximately 580,000 farms?

Line 104 and 105: Suggest changing “farmers’ behavior” to “producers’ practices”.

Line 106: Were there two replicates for each run for a total N =4? That’s not many experimental units.

Line 108: Do you mean just the feces or the manure (mixture of feces and urine)?

Line 151: Maybe I missed it, but how did you determine the volatile solids content?

Line 202: What were the variables in your statistical model?

Line 214-216: Were all the cattle from all the farms fed the same diet? If not, how did you account for differences in nutrient composition of the manure from the cattle fed different diets?

Line 373: You state the increase in humidity may reduce or delay the drying of manure, which may lead to delayed moisture loss and an inhabitation of CH4 emission. However, would higher moisture content increase anaerobic conditions in the manure which would increase CH4 production and emission?

Line 385: Suggest changing to read, “A similar example…”

Line385-387: Beef should be even lower than poultry since poultry manure typically has very high N content compared to beef.

Line 419: Why do you think the composition of the methanogens was not consistent between the two run? How confident are you in your methane data with only N=4 and two runs?

Line 400-401: You measured pH and EC but don’t discuss how these factors may have influenced GHG emissions. You seem to focus only on moisture and temperature.

Line 453-489: This is an interesting discussion that seems loosely tied to this paper. It seem a bit out of place in this particular manuscript.
